# Continuous positive airway pressure for term and ≥34^+0^ weeks’ gestation newborns at birth: A systematic review

**DOI:** 10.1016/j.resplu.2022.100320

**Published:** 2022-11-08

**Authors:** Birju A. Shah, Jorge G. Fabres, Tina A. Leone, Georg M. Schmölzer, Edgardo G. Szyld, International Liaison Committee on Resuscitation Neonatal Life Support Task Force

**Affiliations:** a Department of Pediatrics, College of Medicine, University of Oklahoma Health Sciences Center, Oklahoma City, OK, United States; b Department of Neonatology, School of Medicine, Pontificia Universidad Católica de Chile, Chile; c Columbia University Vagelos College of Physicians and Surgeons, New York City, NY, United States; d University of Alberta, Canada

**Keywords:** CI, confidence interval, CPAP, Continuous Positive Airway Pressure, GRADE, Grading of Recommendations, Assessment, Development and Evaluation, ILCOR, International Liaison Committee on Resuscitation, MD, mean difference, NICU, neonatal intensive care unit, NNT, number needed to treat, PEEP, positive end expiratory pressure, PICO, population, intervention, comparison, outcome, PRISMA, Preferred Reporting Items for Systematic Reviews and Meta-analyses, RCT, randomized controlled trial, RR, risk ratio, Transition after birth, Spontaneously breathing, Term, Late preterm, Infant neonates, Stabilization, At-risk, Respiratory distress, Meta-analysis

## Abstract

**Background:**

Respiratory distress is common during transition after birth, but the effect of continuous positive airway pressure applied in the delivery room has not been systematically evaluated in spontaneously breathing term and ≥34^+0^ weeks’ gestation infants.

We aimed to compare delivery room continuous positive airway pressure with no delivery room continuous positive airway pressure for term and ≥34^+0^ weeks’ gestation newborn infants at birth.

**Methods:**

Information sources: Medline, Embase, Cochrane Databases, Database of Abstracts of Reviews of Effects, and Cumulative Index to Nursing and Allied Health Literature. The Databases were last searched in October 2021.

Eligibility criteria: Randomized, quasi-randomized, interrupted time series, controlled before-after, and cohort studies with English abstracts.

Synthesis of results: Two authors independently extracted data, assessed risk of bias, and certainty of evidence. The main outcome was admission to the neonatal intensive care unit (NICU) or higher level of care receiving any positive pressure support. Data were pooled using fixed effects models.

Risk of bias: Was assessed using the Cochrane Risk of Bias Tool for randomized trials and the Non-Randomized Studies of Interventions Tool (ROBINS-I) for observational studies.

**Results:**

In this meta-analysis, two randomized control trials (323 newborns delivered by cesarean section) showed that delivery room continuous positive airway pressure decreased the likelihood of NICU admission (risk ratio (RR) 95% confidence interval (CI) 0.27 (0.11–0.66), p < 0.005) and NICU respiratory support (RR (95% CI) 0.18 (0.05–0.60), p = 0.005) when compared with no delivery room continuous positive airway pressure. However, in two before-after studies (8,476 newborns), delivery room continuous positive airway pressure use was associated with an increased risk of air leak syndrome when compared with no delivery room continuous positive airway pressure.

**Discussion:**

Certainty of evidence was very low for all outcomes. Among term and ≥34^+0^ weeks’ gestation infants having or at risk of having respiratory distress, there is insufficient evidence to suggest for or against routine use of continuous positive airway pressure in the delivery room.

Funding: No Funding has been received to conduct this study.

**Clinical Trial Registration:** This systematic review has been registered with the International Prospective Register of Systematic Reviews (http://www.crd.york.ac.uk/prospero/) [identifier: CRD42021225812].

## Introduction

At birth, major and complex physiological changes must take place to ensure a successful transition from fetal to neonatal life. The newborn infant must initiate spontaneous breathing, clear fetal lung fluid, and develop a functional residual capacity. When these processes do not occur effectively the infant will develop respiratory distress which is defined as any signs of breathing difficulties.^1^ Respiratory distress affects up to 7% of term (≥37^+0^ weeks’ gestational age) newborn infants after birth, and 9% of late preterm (34^+0^–36^+6^ weeks’ gestation) infants especially those born by elective cesarean section deliveries who may not have the benefit of labor.^2^, ^3^ Respiratory distress is responsible for the majority of neonatal intensive care units (NICU) admissions.^4^ Moreover, 15% of term infants and 29% of late preterm infants admitted to the NICU develop significant respiratory morbidity.^3^

Treatment for respiratory distress traditionally consisted of oxygen given via headbox, low-flow nasal prong or cannula, or face mask. More recently, continuous positive airway pressure (CPAP), a non-invasive form of respiratory support, has become widely used for the prevention and treatment of respiratory distress, with the aim of reducing the need for invasive mechanical ventilation. It applies positive pressure to the airways of spontaneously breathing infants throughout the respiratory cycle. In preterm infants of <33 weeks’ gestation, early CPAP use decreases the need for mechanical ventilation and decreases the risk of death or chronic lung disease.^5^

CPAP has been included in the neonatal resuscitation algorithm for spontaneously breathing infants with labored breathing or persistent cyanosis after the initial steps of resuscitation. For spontaneously breathing preterm newborn infants with respiratory distress requiring respiratory support in the delivery room, the International Liaison Committee on Resuscitation (ILCOR) Neonatal Task Force has suggested initial use of CPAP rather than intubation and intermittent PPV.^6^ Although provision of CPAP in the delivery room for late preterm and term infants has become more popular; this practice has not been systematically evaluated. Therefore, a decision was made by the ILCOR Neonatal Task Force to prioritize this review. The Population, Intervention, Comparator, Outcome (PICO) question was developed and approved by the ILCOR Neonatal Task Force. The aim of this systematic review and meta-analysis was to compare the use of CPAP at different pressures with or without supplemental oxygen with no CPAP with or without supplemental oxygen in spontaneously breathing infants of term and ≥34^+0^ weeks’ gestation with respiratory distress and/or low oxygen saturations during the immediate transition after birth in the delivery room.

## Methods

### Protocol

This systematic review was completed as part of the ILCOR Neonatal Life Support Task Force continuous evidence review process. This review followed the standard methods of the Cochrane Handbook for Systematic Reviews of Interventions V.6.1.^7^ Reporting followed the Preferred Reporting Items for Systematic Reviews and Meta-Analyses (PRISMA) guidelines for meta-analysis in health care interventions.^8^ The protocol was registered with the International Prospective Register of Systematic Reviews (PROSPERO) on January 18, 2021 [CRD42021225812].^9^ There was no review protocol prepared and there were no amendments to information provided at registration or in the protocol.

We searched the following databases through the Ovid interface in November 2020: Cochrane Central Register of Controlled Trials (CENTRAL) (2020, repeat 2021), Medline and Epub Ahead of Print, In-process, In-Data-Review & Other Non-Indexed Citations (1946 to November 2020, repeat October 2021); and Embase (1974 to November 2020, repeat October 2021). The initial searches were conducted by an information specialist in close consultation with the review team. An online appendix includes search strategies detailing the controlled vocabulary and additional terms used to describe each concept, as well as the special features (e.g., limits, explode, focus, etc.) utilized within each database. Additional database searches were conducted in PubMed (for the previous year); Cumulative Index to Nursing and Allied Health Literature (CINAHL) Plus with Full Text via EBSCOhost (1981 to November 2020, repeat October 2021); and Electronic Library Online (SciELO) using a predefined search strategy consistent with Cochrane’s standard search strategy for neonates, in combination with respiratory management terms. We also searched clinical trial registries for ongoing and recently completed trials (November 30, 2020) including the WHO’s International Clinical Trials Registry Platform, ClinicalTrials.gov, International Standard Randomized Controlled Trial Number Registry, European Union Clinical Trials register, and the Australia New Zealand Clinical Trials Registry.

Randomized controlled trials (RCTs), ancillary analyses of RCTs, quasi-randomized controlled studies, cluster RCTs, and non-randomized studies (non-RCTs, interrupted case series, controlled before-and-after studies, cohort or cross-sectional studies) comparing different policies and procedures regarding respiratory management in spontaneously breathing term and ≥34^+0^ weeks’ gestation infants in the delivery room were eligible for inclusion. There were no restrictions on year of publication. There were no language restrictions provided if there was an English abstract.^9^, ^10^ We reviewed the reference lists of all identified articles that were passed through to full text review for relevant articles that were not identified in the primary search. We searched for follow-up reports of included studies by searching for their trial registration number in Medline. Unpublished studies (e.g., conference abstracts or trial protocols), review articles, editorials, comments, letters, news articles, case reports, animal studies, and manikin studies were excluded. Study authors were contacted, when appropriate, to request additional unpublished data.

### Eligible patients

Studies in which authors compared the following interventions in term and ≥34^+0^ weeks’ gestation infants having or at risk of having respiratory distress in the delivery room during the immediate transition after birth were included:

1. Treatment CPAP, defined as application of CPAP in the delivery room only in symptomatic newborns with respiratory distress with or without supplemental oxygen for low oxygen saturations.
  a. Respiratory distress is defined as one or more signs of increased work of breathing such as chest retractions, tachypnea (respiratory rate > 60 breaths per minute), nasal flaring, and/or grunting.
  b. Low oxygen saturations are defined as lower than currently recommended target preductal oxygen saturations.^6^
  c. CPAP included all manner of providing CPAP in the delivery room such as T-piece, bubble CPAP, and flow inflating bag.
2. Prophylactic CPAP, defined as application of delivery room CPAP in newborns without respiratory distress with or without supplemental oxygen.

Studies were eligible if they included at least 1 of the following 2 review comparisons:

I. Treatment CPAP versus no CPAP
II. Prophylactic CPAP versus no CPAP

While the PROSPERO population was defined as spontaneously breathing ≥34 weeks’ gestation newborns with respiratory distress and/or low oxygen saturations during transition after birth, the article population is defined as ≥34 + 0 weeks' gestation and term infants having or at risk of having respiratory distress in the delivery room during the immediate transition after birth and receiving either CPAP as treatment or prophylactically. This adjustment in the population was done as we only identified studies with this adapted patient collective.

### Outcomes

We used the rating of patient-oriented outcomes determined by ILCOR for decision-making in resuscitation for newborns.^11^ The primary outcome was admission to neonatal intensive care unit (NICU) or higher level of care receiving any positive pressure support (important). Secondary outcomes included i) provision of tracheal intubation or chest compressions in the delivery room (important), ii) use and duration of respiratory support in NICU (important), iii) air-leak syndromes including pneumothorax and pneumomediastinum (important), iv) death prior to hospital discharge (critical), v) length of hospital stay (important), vi) moderate to severe neurodevelopmental impairment in early childhood (>18 months, with age-appropriate, validated tools), including cerebral palsy, significant developmental delay (Bayley Scales of Infant Developmental Mental Developmental Index < 70), legal blindness (<20/200 visual acuity), and hearing deficit (aided or < 60 dB on audiometric testing) (critical).^12^

### Study selection

Each title and abstract were independently screened by two authors (BAS and JGF) using Covidence (Covidence Systematic Review Software, Veritas Health Innovation, 2021).^13^ In the event of disagreement at abstract screening, the full text of potentially eligible studies for inclusion was reviewed. Independent reviewers (TAL and EGS) subsequently completed full-text review for eligibility and separately selected studies for inclusion in 2 steps. The full review team resolved disagreements at the full-text stage and final decisions were determined through consensus. The reason for exclusion was specified and captured according to a pre-determined, ordered list of exclusions (i.e., articles including non-invasive positive pressure ventilation, nasal cannula, or CPAP initiated after the delivery room). Inter-rater agreement for article selection was assessed using Cohen’s kappa coefficient at the abstract and full-text stages using Covidence software.

### Data collection, risk of bias, and certainty of evidence assessment

For each study, two authors (BAS and JGF) independently extracted pre-determined study characteristics and study outcomes. If necessary, study authors were contacted to request missing data. Two authors (BAS and JGF) independently assessed risk of bias in individual studies using the Cochrane Risk of Bias Tool^14^ for RCTs and the Risk of Bias in Non-Randomized Studies of Interventions Tool (ROBINS-I)^15^ for observational studies. Risk of Bias for each outcome was summarized across studies. Similarly, two authors (BAS and JGF) assessed the certainty of evidence (confidence in the estimate of effect) for each outcome using Grading of Recommendations, Assessment, Development, and Evaluation (GRADE) framework.^16^ The optimal information size was used to assess imprecision. Imprecision was considered present if the total number of study participants included was less than the optimal information size for the outcome under consideration.^17^ The Risk of Bias and GRADE assessments were evaluated by the full review team to reach consensus and ensure consistency. The evidence profile tables were presented and discussed with the ILCOR Neonatal Life Support Task Force and content experts.

### Data analysis

GRADEPro^18^ (GRADEpro Guideline Development Tool, McMaster University and Evidence Prime, 2021) and Review Manager^19^ (RevMan 5.4.1, The Cochrane Collaboration, 2020) were used to analyze the data. Based on Cochrane recommendations, meta-analyses were performed if ≥2 studies were available.^20^ Observational studies were analyzed and reported if fewer than 2 RCTs/quasi-RCTs provided outcome data or if the certainty of evidence from these RCTs/quasi-RCTs was very low. For dichotomous outcomes, pooled unadjusted risk ratios (RRs) and corresponding 95% confidence intervals (CIs) were reported using the Mantel-Haenszel fixed effects method. The pooled absolute effect difference and number needed to treat (NNT) were calculated when the RR revealed a statistically significant difference. Pooled continuous variables were reported as weighted mean differences (MDs) and corresponding 95% CIs using the Inverse Variance fixed effect method. Forest plots were used for graphical representation of RRs and MDs. Heterogeneity was measured using the I^2^ statistic.^21^ Substantial heterogeneity was considered present if the I^2^ statistic was >50%. If pooled analysis was judged inappropriate, individual studies were analyzed and interpreted separately. If there was evidence of clinical heterogeneity, we tried to explain this on the basis of the different study characteristics and/or subgroup analyses.

Prespecified subgroup analyses were planned if >2 studies were available with relevant outcome information related to mode of delivery (vaginal versus cesarean section deliveries), gestational age (34^+0^–36^+6^ weeks versus ≥37^+0^ weeks), any previous positive pressure support or not, supplemental oxygen or not, interface of support (facemask versus nasal prongs), device of support (T-piece versus flow-inflating bag), and level of CPAP (4–6 cm H_2_O versus >6 cm H_2_O).

## Results

### Literature search and study selection

**Fig. 1:**
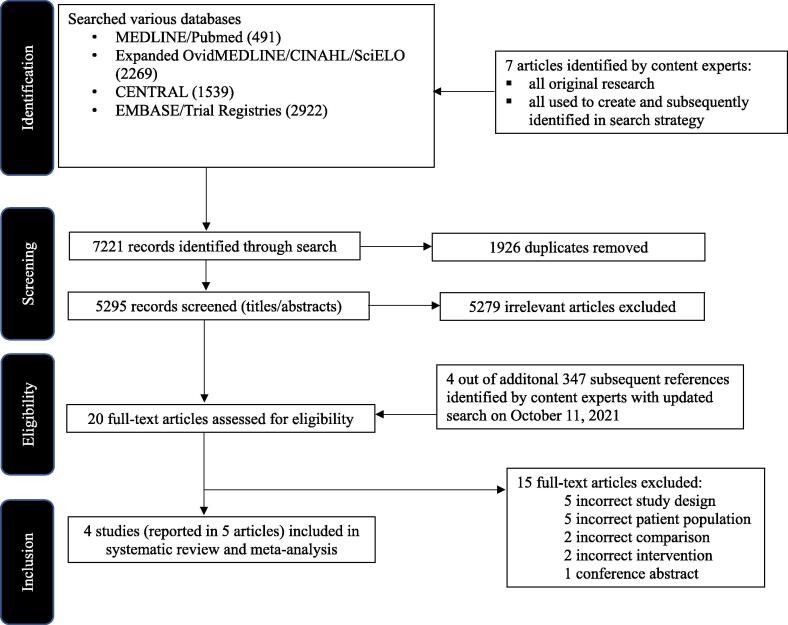
PRISMA flow diagram of study selection.

The initial search strategy identified 7,221 records; after removing 1,926 duplicates, 5,295 titles and abstracts were screened. A total of 20 full-text articles were assessed for eligibility. Fig. 1 presents the PRISMA study selection diagram. The Cohen’s kappa coefficient was 0.75 at the abstract stage and 1.0 at the full-text stage.

An updated search was performed on October 8, 2021, which yielded an additional 347 references. Four further studies were identified for full text evaluation, however none met inclusion criteria (Fig. 1).

### Study characteristics

The systematic review identified two RCTs^22^, ^23^ and two observational, before-after studies, one of which was divided into two publications.^24^, ^25^, ^26^ Additional data were obtained from one author and included in this meta-analysis.^24^, ^25^ Characteristics of included studies are reported in Table 1.

**Table 1:** Study characteristics.

Study		Study Characteristics				Total Patients (n)	Patients by Respiratory Intervention (n)		Outcomes						
		Years of Recruitment	Country	Multi or Single Center	Study Design		CPAP	No CPAP	Death	NDI	NICU Admission	Other Respiratory Outcomes		DR Interventions (ETT/ CC)	Length of Hospital Stay
												NICU Respiratory Support	Air-leak Syndrome		
Celebi	2016	2012–13	Turkey	Single	RCT	259	134	125	+	−	+	+	+	−	−
Osman	2019	2016–17	Egypt	Single	RCT	78	39	38*	+	−	+	+	+	−	+
Hishikawa	2016	2008–12	Japan	Single	Obs	1563	328	1235	−	−	+	+	+	−	+
Smithhart	2019	2005–15	USA	Single	Obs	6913	1001	5912	−	−	−	−	+	−	−

Abbreviations: RCT, Randomized Controlled Trial; Obs, Observational study; CPAP, Continuous Positive Airway Pressure; NDI, Neurodevelopmental Impairment; NICU, Neonatal Intensive Care Unit; DR, Delivery Room; ETT, Endotracheal Tube; CC, Chest Compressions.

* One patient did not receive allocated intervention due to diaphragmatic hernia.

A total of 8,799 patients, 323 from RCTs^22^, ^23^ and 8,476 from observational cohorts^25^, ^26^ were included. Both RCTs were single-center trials and conducted in Egypt and Turkey respectively.^22^, ^23^ The observational studies were single-center and conducted in Japan and United States of America respectively.^25^, ^26^ The definition of “respiratory distress” varied across studies, and included at least one of following signs of increased work of breathing: chest retractions, tachypnea (respiratory rate > 60 breaths per minute), nasal flaring, and/or grunting.

### Patient characteristics

Table 2 shows patient characteristics of the included studies. All of the included studies utilized face mask to deliver CPAP in the delivery room. Both RCTs included only infants born by cesarean section deliveries, one study by Celebi et al used early prophylactic CPAP and the other study by Osman et al used treatment CPAP.^22^, ^23^ One observational study by Hishikawa et al included only term newborn infants; while the other observational study reported delivery room CPAP data in the nested cohort which included only newborn infants admitted to NICU.^25^, ^26^

**Table 2:** Patient characteristics.

Study		Respiratory Intervention (N)		Gestational Age, Weeks (Mean ± SD)	Male Sex, N (%)	Birth Weight, Grams (Mean ± SD)	Cesarean Delivery, N (%)
Celebi	2016	Prophylactic CPAP	134	34–36 weeks: n = 15	68 (50.7)	3294 ± 491	134 (100)
				37–38 weeks: n = 119			
		No prophylactic CPAP	125	34–36 weeks: n = 20	63 (50.4)	3190 ± 457	125 (100)
				37–38 weeks: n = 115			
Osman*	2019	Treatment CPAP	34	37.6 ± 0.7	17 (50)	3040 ± 420	34 (100)
		No treatment CPAP	30	37.7 ± 0.7	16 (60)	3070 ± 440	20 (100)
Hishikawa	2016	Treatment CPAP	328	38.7 ± 1.1	171 (52)	3003 ± 374	87 (26.5)
		No treatment CPAP	1235	38.7 ± 1.1	629 (51)	3000 ± 386	352 (28.5)
Smithhart	2019	Treatment CPAP	1001	38 ± 2	585 (58)	3159 ± 669	657 (66)
		No treatment CPAP	5912	38 ± 2	3150 (53)	3123 ± 729	3124 (53)

CPAP, continuous positive airway pressure; SD, standard deviation.

* Five participants from CPAP group excluded (3 culture-proven sepsis, 2 congenital heart disease) and 8 participants from no CPAP group excluded (4 culture-proven sepsis, 3 congenital heart disease, 1 persistent pulmonary hypertension).

### Risk of Bias

Both RCTs were considered to have a high risk of bias for non-blinding of participants/personnel (Table 3).^22^, ^23^ In the study by Celebi et al, the risk of bias was assessed as high due to lack of blinding of outcome assessors, and this was unclear in study by Osman et al.^22^, ^23^ In both RCTs, the risk of bias was assessed unclear for allocation concealment and high for other sources of bias due to various reasons including lack of block randomization in the study by Celebi et al and no protocol criteria for NICU admissions in the study by Osman et al.^22^, ^23^.

**Table 3:** Risk of Bias for Randomized Controlled Trials. Cochrane risk of bias for RCTs.

Study	Sequence Generation	Allocation Concealment	Blinding of Participant Personnel	Blinding of Outcome Assessors	Incomplete Outcome Data	Selective Outcome Reporting	Other Sources of Bias	Overall Bias
Celebi 2016	Low	Unclear	High (a)	High (a)	Low	Low	High (b)	High
Osman 2019	Low	Unclear	High (a)	Unclear	Low	Low	High (c)	High

(a) not blinded; (b) lack of block randomization; (c) no study protocol or pre-defined criteria for primary outcome.

The observational study by Hishikawa et al was determined to have a moderate overall risk of bias due to serious risk of bias in measurement of outcomes.^25^ The other observational study by Smithhart et al was determined to have a high overall Risk of Bias due to serious Risk of Bias in selection of participants, deviation from intended interventions, and selection of reported results (Table 4).^26^

**Table 4:** Risk of Bias for Observational Studies, ROBINS-I cohort observational risk of bias.

Study	Bias due to Confounding	Bias due to Selection of Participants	Bias in Classification of Interventions	Bias due to Deviations from Intended Interventions	Bias due to Missing Data	Bias in Measurement of Outcomes	Bias in Selection of Reported Results	Overall Bias
Hishikawa 2016	Moderate	Moderate	Moderate	Moderate	Moderate	Serious (a)	Moderate	Moderate
Smithhart 2019	Moderate	Serious (b)	Moderate	Moderate	Moderate	Moderate	Serious (b,c)	Serious

(a) only symptomatic newborns; (b) only newborns admitted to NICU; (c) spontaneous pneumothorax may impact denominator.

### Certainty of evidence

All outcomes selected for GRADE analysis were rated as very low certainty of evidence because of serious risk of bias, inconsistency, indirectness, and/or imprecision (Table 5).

**Table 5:** Summary of GRADE assessment.

Outcome	Certainty Assessment							Number of Patients		Effect		Certainty	Importance
	Number of Studies	Study Design	Risk of Bias	Inconsistency	Indirectness	Imprecision	Other Considerations	CPAP	No CPAP	Relative (95% CI)	Absolute (95% CI)		
NICU admission	2	RCTs	Serious^a^	Not serious	Serious^b^	Serious^c^	None	6/168 (3.6%)	20/155 (12.9%)	RR 0.27 (0.11–0.36)	94 fewer per 1,000 (from 115 fewer to 44 fewer)	⊕◯◯◯ Very low	Important
	1	Observation study	Serious^d^	Not serious	Serious^b^	Serious^c^	None	63/328 (19.2%)	67/1235 (5.4%)	RR 3.54 (2.57–4.88)	138 more per 1,000 (from 85 more to 210 more)	⊕◯◯◯ Very low	Important
NICU respiratory support	2	RCTs	Serious^a^	Not serious	Serious^b^	Serious^c^	None	3/168 (1.8%)	15/155 (9.7%)	RR 0.18 (0.05–0.60)	79 fewer per 1,000 (from 92 fewer to 39 fewer)	⊕◯◯◯ Very low	Important
	1	Observation study	Serious^d^	Not serious	Serious^e^	Serious^c^	None	31/328 (9.5%)	15/1235 (1.2%)	RR 7.78 (4.25–14.24)	84 more per 1,000 (from 39 more to 161 more)	⊕◯◯◯ Very low	Important
Pulmonary air-leak syndrome	2	RCTs	Serious^a^	Not serious	Serious^g^	Serious^h^	None	0/168 (0%)	0/155 (0%)	Not estimable		⊕◯◯◯ Very low	Important
	2	Observation studies	Very serious^i^	Seriousj	Serious^k^	Not serious	None	213/1329 (16%)	243/7147 (3.4%)	RR 4.92 (4.13–5.87)	133 more per 1,000 (from 106 more to 166 more)	⊕◯◯◯ Very low	Important
Length of hospital stay	1	RCT	Serious^l^	Not serious	Serious^m^	Serious^c^	None	34	30	-	MD 0.8 days lower (1.65 lower to 0.05 lower)	⊕○○○ Very low	Important
	1	Observational study	Serious^d^	Not serious	Serious^e^	Serious^n^	None	328	1235	-	MD 1 day higher (0.31 higher to 1.69 lower)	⊕○○○ Very low	Important
Death prior to hospital discharge	2	RCTs	Serious^a^	Not serious	Serious^b^	Serious^o^	None	0/168 (0%)	1/155 (0.6%)	RR 0.30 (0.01–6.99)	5 fewer per 1,000 (from 6 fewer to 39 more)	⊕○○○ Very low	Critical
Tracheal intubation or chest compression in the delivery room – NOT REPORTED	–	–	–	–	–	–	–	–	–	–	–	–	Important
Neurodevelopmental impairment at ≥18 months – NOT REPORTED	–	–	–	–	–	–	–	–	–	–	–	–	Critical

**CI,** confidence interval; **MD,** mean difference; **RR,** risk ratio.

^f^Although 95% CI is narrow and does not cross 1.0 for RR, the total number of patients for this outcome is less than optimal information size (OIS).^17^

^j^I^2^ = 68%.^21^

a Both included studies (Celebi 2016, Osman 2019) were judged to have overall serious risk of bias for this primary outcome due to concerns related to lack of blinding and apparent variation in thresholds for NICU admission with no pre-specified criteria for NICU admission.

b One study (Celebi 2016) applied CPAP to all babies regardless of signs of respiratory distress and both studies (Celebi 2016, Osman 2019) enrolled only newborns delivered by cesarean section.

c Although 95% CI is narrow and does not cross 1.0 for RR, the total number of patients for this outcome is less than optimal information size (OIS).

d The single included study (Hishikawa 2016) was judged to have overall serious risk of bias for this primary outcome due to concern related to.

e One included study (Hishikawa 2016) involved only term newborns.

g Variable definitions of respiratory distress were used and etiology of respiratory distress was judged to be heterogenous.

h No events recorded in either group. The total number of patients for this outcome is less than OIS.

i Two included studies (Hishikawa 2016, Smithhart 2019) were judged to have overall very serious risk of bias for this outcome due to significant selection bias and one included study (Smithhart 2019) involved only newborns admitted to NICU.

k The method of determining the presence of pulmonary air-leak was indirect (chest radiograph was only obtained if clinical symptoms were identified). Spontaneous pneumothorax may impact denominator.

l The single included study (Osman 2019) was judged to have overall serious risk of bias for this outcome.

m The single included study (Osman 2019) enrolled only newborns delivered by cesarean section.

n The total number of patients for this outcome is less than OIS.

### Outcome analysis

#### Primary outcome – NICU admission

**Fig. 2:**
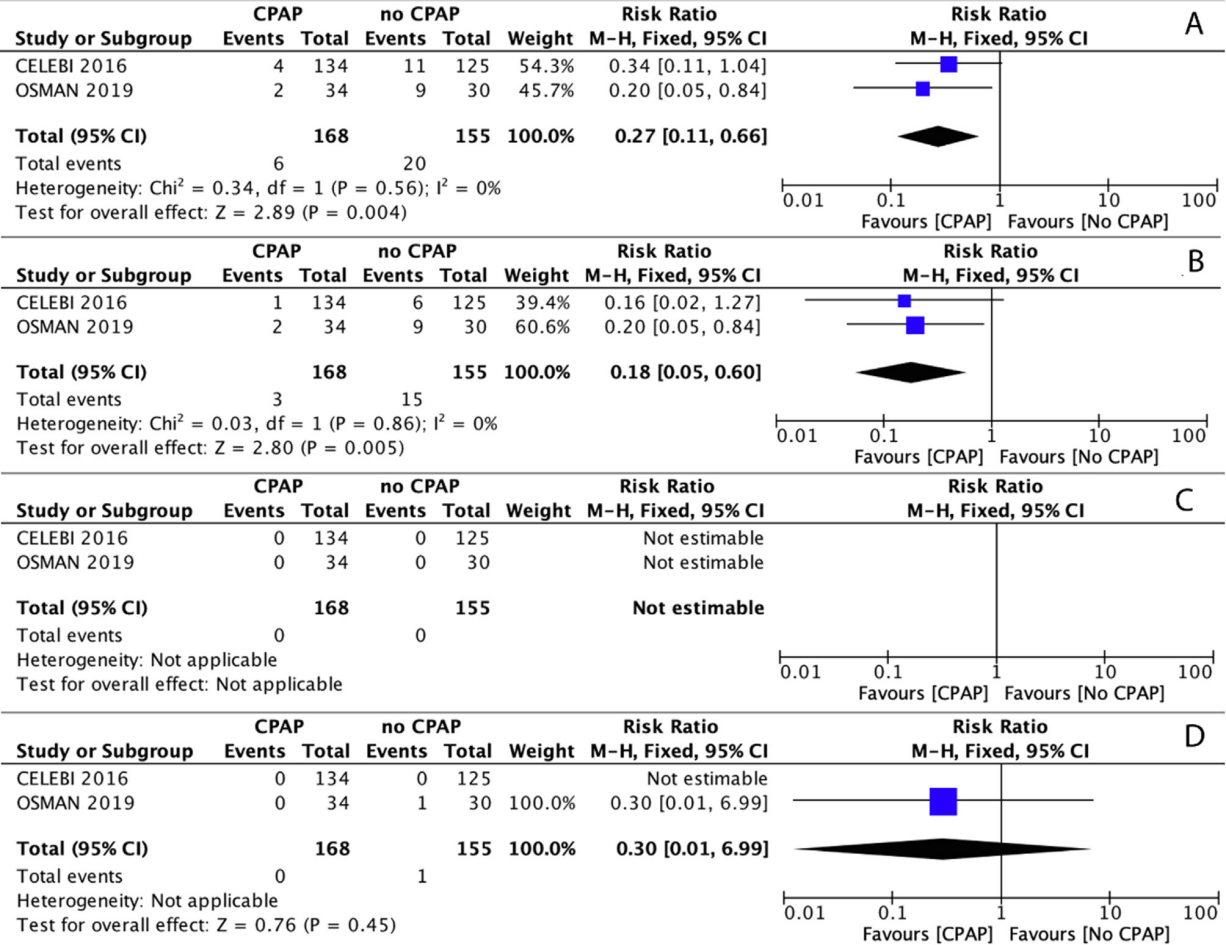
Summary of Results from Randomized Controlled Trials: Continuous Positive Airway Pressure (CPAP) compared with no CPAP in labor and delivery room among term and late preterm newly born infants born by cesarean delivery with or without respiratory distress. (A) neonatal intensive care unit (NICU) admissions. (B) NICU respiratory support. (C) pulmonary air-leak syndromes. (D) death prior to hospital discharge. Abbreviations: M−H = Mantel-Haenszel method; IV = Inverse Variance method; Fixed = fixed effects; CI = confidence intervals; CPAP = continuous positive airway pressure.

In two RCTs enrolling 323 term and ≥34^+0^ weeks’ gestation infants born by cesarean section deliveries with or without respiratory distress, the pooled estimate for NICU admissions was significantly lower for the group receiving prophylactic and/or treatment CPAP compared with the group receiving no CPAP (RR (95% CI) 0.27 (0.11–0.66); absolute effect (95% CI) 94 fewer (115 fewer to 44 fewer) per 1,000; NNT (95% CI) 11 (9–23); very low Certainty of Evidence) (Fig. 2).^22^, ^23^

In the observational study by Hishikawa et al involving 1,563 term newborn infants, delivery room CPAP treatment was associated with an increased risk of NICU admission when compared with no CPAP (RR (95% CI) 3.54 (2.57–4.88); absolute effect (95% CI) 138 more (85 more to 210 more) per 1,000; very low Certainty of Evidence).^25^

#### Secondary outcomes

##### NICU respiratory support

In two RCTs, the need for NICU respiratory support was significantly reduced in the group receiving CPAP compared with the group receiving no CPAP (RR (95% CI) 0.18 (0.05–0.6); absolute effect (95% CI) 79 fewer (92 fewer to 39 fewer) per 1,000; NNT (95% CI) 13 (11–26); very low Certainty of Evidence) (Fig. 2).^22^, ^23^

In the observational study by Hishikawa et al involving 1,563 term newborn infants, CPAP treatment was associated with an increased need for respiratory support in NICU when compared with no CPAP (RR (95% CI) 7.78 (4.25–14.24); absolute effect (95% CI) 82 more (39 more to 161 more) per 1,000; very low Certainty of Evidence).^25^

##### Air leak syndrome

**Fig. 3:**
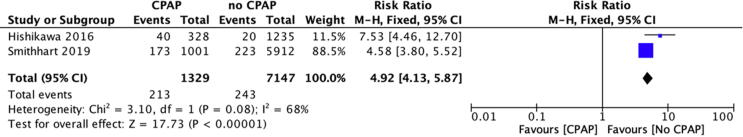
Summary of Results from Observational Studies: Continuous Positive Airway Pressure (CPAP) compared with no CPAP in labor and delivery room among term and late preterm newly born infants with respiratory distress: pulmonary air-leak syndromes. Abbreviations: M−H = Mantel-Haenszel method; IV = Inverse Variance method; Fixed = fixed effects; CI = confidence intervals; CPAP = continuous positive airway pressure.

In the two observational studies involving 8,476 term and ≥34^+0^ weeks’ gestation infants, CPAP treatment was associated with an increased risk of pulmonary air leak syndrome when compared with no CPAP (RR (95% CI) 4.92 (4.13–5.87); absolute effect (95% CI) 133 more (106 more to 166 more) per 1,000; very low Certainty of Evidence) (Fig. 3).^25^, ^26^

No air leaks were reported in either RCT (Fig. 2).^22^, ^23^

##### Length of hospital stay

The RCT by Osman et al enrolling 64 term and ≥34^+0^ weeks’ gestation infants, reported no significant difference in length of hospital stay between groups (MD (95% CI) 0.8 days lower (1.65 days lower to 0.05 days higher); very low Certainty of Evidence).^23^

In the observational study by Hishikawa et al involving 1,563 term newborn infants, the length of hospital stay was higher in CPAP-treated infants when compared with no CPAP (MD (95% CI) 1 day higher (0.31 days higher to 1.69 days higher); very low Certainty of Evidence).^25^

##### Death prior to hospital discharge

The pooled estimate for death prior to hospital discharge was not significantly different for the group receiving CPAP compared with the group receiving no CPAP (RR (95%CI) 0.3 (0.01–6.99); absolute effect (95% CI) 5 fewer (6 fewer to 39 more) per 1,000; very low Certainty of Evidence) (Fig. 2).^22^, ^23^

No data were reported for tracheal intubation or chest compressions in the delivery room, or neurodevelopmental impairment at ≥18 months of age.

### Subgroup analyses

The available data were insufficient to perform any prespecified subgroup analyses.

## Discussion

A growing proportion of term and ≥34^+0^ weeks’ gestation infants require admission to the NICU due to respiratory distress.^27^, ^28^ Identifying the most effective strategy to aerate the lungs at birth, maintain functional residual capacity and support breathing is a research priority. CPAP applied immediately after birth in preterm infants of <33 weeks’ gestation improves survival without bronchopulmonary dysplasia.^5^ This knowledge has been extrapolated to term and ≥34^+0^ weeks’ gestation infants despite limited evidence. To our knowledge, this is the first systematic review and meta-analysis evaluating delivery room CPAP in this population. Our analysis of data from 2 RCTs, demonstrated that term and ≥34^+0^ weeks’ gestation infants born by cesarean section deliveries who receive CPAP compared with no CPAP may be admitted less often to the NICU.^22^, ^23^ However, two cohort studies reported delivery room CPAP use was associated with an increased risk of pulmonary air leak syndromes in term and ≥34^+0^ weeks’ gestation newborn infants with respiratory distress.^25^, ^26^

During transition after birth, aeration of the lungs results in lung liquid clearance, establishes functional residual capacity and increases pulmonary blood flow. CPAP applies positive pressure to the airways of a spontaneously breathing baby throughout the respiratory cycle. When term and ≥34^+0^ weeks’ gestation infants are born by cesarean section deliveries, they commonly have higher pulmonary liquid to air ratios, which predisposes them to respiratory distress.^29^ Applying positive end-expiratory pressure has been shown to prevent airway liquid re-entry during expiration in newborn rabbits with elevated pulmonary liquid to air ratios.^30^ Delayed fluid clearance contributes to the pathophysiology of respiratory distress. Supporting an infant’s respiratory efforts with CPAP may attenuate secondary lung injury by avoiding atelectotrauma, secondary surfactant deficiency, oxygen-related injury, and pulmonary hypertension.^31^ CPAP prevents airway collapse, decreases intrapulmonary shunt, and facilitates and maintains alveolar recruitment.^32^

However, higher levels of airway pressures may lead to over-distention resulting in air leak syndromes.^33^ This may be due to combination of positive pressures applied via CPAP and pressures generated by the infant during spontaneous breathing. Since the pressure required to open the lung is higher than that required to maintain aeration, prolonged exposure to higher than needed positive airway pressure in transitioning newborns with improving lung compliance may lead to air leaks.^31^ Morley et al reported a higher incidence of pneumothorax in the COIN trial with CPAP starting at 8 cm H_2_O in preterm infants of 25^+0^–28^+6^ weeks’ gestation.^34^ Higher lung compliance and lower chest compliance may contribute to pneumothorax with CPAP in term and ≥34^+0^ weeks’ gestation infants when compared with those in preterm infants.^35^, ^36^ Smithhart et al described a positive association between CPAP use and pneumothorax that was more significant when CPAP was provided without supplemental oxygen.^26^ This has been speculated to be due to differences in compliance.

The differences in outcomes between the RCTs and observational studies may be due to several factors.^22^, ^23^, ^25^, ^26^ All studies used a facemask as an interface during delivery room care, which may increase the dead space and lead to insufficient ventilation, but does not explain the differences between studies.^37^ Differences in rates of pulmonary air-leaks among included studies may be related to differential effect of NICU care and respiratory support with CPAP in two observational studies^25^, ^26^ when compared with two randomized trials.^22^, ^23^ Alternatively, the differences between RCTs and observational studies such as earlier timing of initiation and limited duration (20-minute) of CPAP in two RCTs may explain the variability. Additionally, the subjects treated in the different studies may be inherently different due to differences in selection of included infants in each study. These studies may also be different from studies in preterm infants because the etiology of respiratory distress among term and ≥34^+0^ weeks’ gestation newborn infants is heterogeneous and includes transient tachypnea of newborn, respiratory distress syndrome (surfactant deficiency), pneumonia, meconium aspiration syndrome and spontaneous pneumothorax.

The strengths of this systematic review include a comprehensive literature search performed by an information specialist with refined search strategy including an updated search, use of standardized methods of systematic review and meta-analysis with assessment of Risk of Bias for each outcome, and review by an international group of neonatal resuscitation content experts. However, the findings of this review should be used with caution because i) different definitions of respiratory distress were used in the included studies; ii) only studies with English abstracts were included; iii) Bayesian analysis was not performed, as standardized methods of the ILCOR systematic review were followed;^7^ iv) rates or proportions from one observation study^26^ were among infants admitted to NICU and not all newborns; and v) the risk of type 2 error for detecting both benefits and harms, as the optimal information size was reached for only one outcome despite nearly 8,800 newborns included in our review.

Since respiratory distress in term or ≥34^+0^ weeks’ gestation infants encompass varied and dynamic pathophysiology, more RCTs are needed regarding whether there are subgroups of term and ≥34^+0^ weeks’ gestation infants having or at risk of having respiratory distress who may benefit from delivery room CPAP and others who may derive no benefit or possible harm. Hence, the response to delivery room CPAP in a newborn with meconium aspiration, a newborn with undiagnosed congenital diaphragmatic hernia, or an ≥34^+0^ weeks’ gestation infant with respiratory distress born by scheduled cesarean section deliveries may be different. Persistent gaps in knowledge include the impact of labor or antenatal corticosteroids on clinical outcomes when CPAP is used for respiratory distress in the delivery room, the effect of CPAP in 34^+0^ – 36^+6^ weeks’ versus ≥37^+0^ weeks’ gestation populations, and the effect of CPAP on newborns delivered vaginally. Additionally, future studies should address the effects of various interfaces and devices used to apply CPAP, higher versus lower levels of CPAP, and differential effects of supplemental oxygen when CPAP is used for respiratory distress in the delivery room.

## Conclusion

For spontaneously breathing term and ≥34^+0^ weeks’ gestation newborn infants having or at risk of having respiratory distress in the delivery room, there is insufficient published evidence to suggest for or against routine use of CPAP compared with no CPAP. Large multicenter high quality RCTs evaluating the effect of delivery room CPAP for ≥34^+0^ weeks’ gestation and term newborn infants are needed.

## Conflict of Interest Disclosures (includes financial disclosures)

The authors have no conflicts of interest relevant to this article to disclose.

## Funding/support

No funding was secured for this study.

## Article summary

This systematic review compares CPAP with no CPAP in spontaneously breathing term and ≥34^+0^ weeks’ gestation infants in the delivery room.

## Contributor’s statement

Drs. Shah and Fabres screened studies, abstracted data, completed risk-of-bias and GRADE evaluations, carried out the initial analyses, and drafted the initial manuscript.

Drs. Shah, Leone, and Szyld prepared the protocol, screened studies, coordinated and supervised data collection, and reviewed and revised the manuscript.

Dr. Schmölzer reviewed the protocol, reviewed the analysis, and critically reviewed the manuscript for important intellectual content.

All authors approved the final manuscript as submitted and agree to be accountable for all aspects of the work.

## CRediT Author Contribution Statement

**Birju A. Shah:** Data curation, Formal analysis, Investigation, Methodology, Resources, Software, Validation, Writing – original draft, Writing – review & editing. **Jorge G. Fabres:** Data curation, Formal analysis, Investigation, Methodology, Resources, Software, Validation, Writing – original draft, Writing – review & editing. **Tina A. Leone:** Data curation, Formal analysis, Investigation, Methodology, Resources, Software, Validation, Writing – original draft, Writing – review & editing. **Georg M. Schmölzer:** Data curation, Formal analysis, Investigation, Methodology, Resources, Software, Validation, Writing – original draft, Writing – review & editing. **Edgardo G. Szyld:** Data curation, Formal analysis, Investigation, Methodology, Resources, Software, Validation, Writing – original draft, Writing – review & editing.

## Appendix 1

Besides the authors Jorge G. Fabres and Georg M. Schmölzer, members of the International Liaison Committee on Resuscitation Neonatal Life Support Task Force include: Myra H. Wyckoff, Helen G. Liley, Peter G. Davis, Jonathan Wyllie, Gary M. Weiner, Yacov Rabi, Vishal S. Kapadia, Jeffrey M. Perlman, Elizabeth E. Foglia, Joe Fawke, Firdose L. Nakwa, Viraraghavan V. Ramaswamy, Walid El Naggar, Ruth Guinsburg, Tetsuya Isayama, Daniela T. Costa-Nobre, Shigeharu Hosono, Maria Fernanda de Almeida, Mandira Daripa Kawakami, Han-Suk Kim, Takahiro Sugiura, R. John Madar, Christopher J.D. McKinlay, Henry C. Lee, Daniele Trevisanuto, Mario Rüdiger, and Charles C. Roehr.
