# Supplementary material for: Continuous positive airway pressure for term and ≥34+0 weeks’ gestation newborns at birth: A systematic review
Source: Resusc Plus. 2022 Nov 8;12:100320. doi: 10.1016/j.resplu.2022.100320 (PMC9649384; doi:10.1016/j.resplu.2022.100320)
Supplement: Supplementary data 1 [file mmc1.docx]

**Appendix**

**Preliminary & Expanded Search** (MEDLINE)

**Search Executed:** November 9, 2020

**Search Set Forwarded for Review:** #33 (Sets forwarded previously on 10/28 🡪 #30 and #32)

**Database:** Ovid MEDLINE(R) and Epub Ahead of Print, In-Process & Other Non-Indexed Citations and Daily <1946 to November 06, 2020>

**Search Strategy:**

--------------------------------------------------------------------------------

1 Continuous Positive Airway Pressure/ (7320)

2 (cpap or ncpap).mp. (9480)

3 (contin$ positiv$ air$ pressur$ or contin$ positiv$ pressur$ or contin$ disten$ air$ pressur$ or contin$ positiv$ trans$ pressur$ or contin$ inflat$ pressur$ or contin$ negat$ disten$ pressur$ or contin$ negat$ pressur$ or contin$ air$ pressur$ or contin$ disten$ pressur$).mp. (13689)

4 or/1-3 (15489)

5 (infan$ or neonat$ or neo-nat$ or newborn$ or new$ born$ or baby$ or babies).mp. (1532706)

6 4 and 5 (3373)

7 limit 6 to animals (158)

8 6 not 7 (3215)

9 remove duplicates from 8 (3187)

10 limit 9 to (case reports or comment or editorial or letter or news) (427)

11 9 not 10 (2760)

12 Term Birth/ (2997)

13 ((term or fullterm$ or full$ term$ or late$ preterm$ or near$ term$) adj (birth$ or childbirth$ or infant$ or neonat$ or neo-nat$ or newborn$ or new$ born$ or baby$ or babies)).mp. (26197)

14 ((34$ or 35$ or 36$ or 37$ or 38$ or 39$ or 40$ or 41$ or 42$) adj2 (gestat$ or week$ or ag$)).mp. (277660)

15 or/12-14 (298979)

16 11 and 15 (491)

17 ((term or fullterm$ or full$ term$ or late$ preterm$ or near$ term$) adj (birth$ or childbirth$ or infant$ or neonat$ or neo-nat$ or newborn$ or new$ born$ or baby$ or babies)).ti. (6307)

18 ((34$ or 35$ or 36$ or 37$ or 38$ or 39$ or 40$ or 41$ or 42$) adj2 (gestat$ or week$ or ag$)).ti,kf. (5549)

19 delivery rooms/ (1612)

20 ((deliver$ or childbirth$ or birth$) adj2 room$).ti,kf. (942)

21 or/12,17-20 (16199)

22 16 and 21 (91)

32 16 not 22 (400)

33 11 not 16 (2269)

***************************

**Search Update** (MEDLINE)

**Search Executed:** October 8, 2021

**Search Set Forwarded for Review:** #14

**Database:** Ovid MEDLINE(R) and Epub Ahead of Print, In-Process, In-Data-Review & Other Non-Indexed Citations and Daily <1946 to October 07, 2021>

**Search Strategy:**

--------------------------------------------------------------------------------

1 Continuous Positive Airway Pressure/ (8060)

2 (cpap or ncpap).mp. (10170)

3 (contin$ positiv$ air$ pressur$ or contin$ positiv$ pressur$ or contin$ disten$ air$ pressur$ or contin$ positiv$ trans$ pressur$ or contin$ inflat$ pressur$ or contin$ negat$ disten$ pressur$ or contin$ negat$ pressur$ or contin$ air$ pressur$ or contin$ disten$ pressur$).mp. (14700)

4 or/1-3 (16594)

5 (infan$ or neonat$ or neo-nat$ or newborn$ or new$ born$ or baby$ or babies).mp. (1588368)

6 4 and 5 (3630)

7 limit 6 to animals (167)

8 6 not 7 (3463)

9 remove duplicates from 8 (3431)

10 limit 9 to (case reports or comment or editorial or letter or news) (460)

11 9 not 10 (2971)

12 ..l/ 11 yr="2021" (212)

13 ..l/ 11 ed=20201028-20211008 (227)

**14 12 or 13 (347)**

***************************

**Additional Database Searches** (Embase | Cochrane Central Register of Controlled Trials)

**Search Executed:** November 24, 2020

**Search Set Forwarded for Review:** #28

**Database:** Embase Classic+Embase <1947 to 2020 November 23>

**Search Strategy:**

--------------------------------------------------------------------------------

1 positive end expiratory pressure/ (57700)

2 (cpap or ncpap).mp. (18714)

3 (contin$ positiv$ air$ pressur$ or contin$ positiv$ pressur$ or contin$ disten$ air$ pressur$ or contin$ positiv$ trans$ pressur$ or contin$ inflat$ pressur$ or contin$ negat$ disten$ pressur$ or contin$ negat$ pressur$ or contin$ air$ pressur$ or contin$ disten$ pressur$).mp. (16659)

4 or/1-3 (62856)

5 (infan$ or neonat$ or neo-nat$ or newborn$ or new$ born$ or baby$ or babies).mp. (1640283)

6 4 and 5 (10785)

7 limit 6 to animals (319)

8 6 not 7 (10466)

9 limit 8 to conference abstracts (1607)

10 8 not 9 (8859)

11 limit 10 to (conference paper or editorial or letter or note) (930)

12 10 not 11 (7929)

13 case report/ (2658446)

14 12 not 13 (6096)

15 term birth/ (3786)

16 ((term or fullterm$ or full$ term$ or late$ preterm$ or near$ term$) adj (birth$ or childbirth$ or infant$ or neonat$ or neo-nat$ or newborn$ or new$ born$ or baby$ or babies)).mp. (36303)

17 ((34$ or 35$ or 36$ or 37$ or 38$ or 39$ or 40$ or 41$ or 42$) adj2 (gestat$ or week$ or ag$)).mp. (470133)

18 delivery room/ (3511)

19 ((deliver$ or childbirth$ or birth$) adj2 room$).mp. (5280)

20 or/15-19 (503724)

21 14 and 20 (1230)

22 *positive end expiratory pressure/ (17039)

23 (cpap or ncpap).ti,kw. (6294)

24 (contin$ positiv$ air$ pressur$ or contin$ positiv$ pressur$ or contin$ disten$ air$ pressur$ or contin$ positiv$ trans$ pressur$ or contin$ inflat$ pressur$ or contin$ negat$ disten$ pressur$ or contin$ negat$ pressur$ or contin$ air$ pressur$ or contin$ disten$ pressur$).ti,kw. (6971)

25 or/22-24 (20479)

26 14 and 25 (2170)

27 21 or 26 (3021)

**28 remove duplicates from 27 (2922)**

***************************

**Search Executed:** November 30, 2020

**Search Set Forwarded for Review:** #7

**Database:** EBM Reviews - Cochrane Central Register of Controlled Trials <October 2020>

**Search Strategy:**

--------------------------------------------------------------------------------

1 Continuous Positive Airway Pressure/ (1063)

2 (cpap or ncpap).mp. (4946)

3 (contin$ positiv$ air$ pressur$ or contin$ positiv$ pressur$ or contin$ disten$ air$ pressur$ or contin$ positiv$ trans$ pressur$ or contin$ inflat$ pressur$ or contin$ negat$ disten$ pressur$ or contin$ negat$ pressur$ or contin$ air$ pressur$ or contin$ disten$ pressur$).mp. (4272)

4 or/1-3 (5937)

5 (infan$ or neonat$ or neo-nat$ or newborn$ or new$ born$ or baby$ or babies).mp. (80288)

6 4 and 5 (1556)

**7 remove duplicates from 6 (1463)**

***************************
